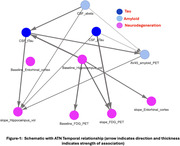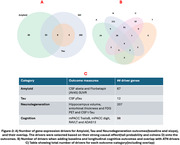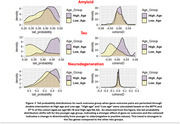# Investigating the ATN (Amyloid, Tau, Neurodegeneration) framework in Alzheimer’s Disease and its causal genetic‐drivers using Digital‐Twins

**DOI:** 10.1002/alz.091647

**Published:** 2025-01-09

**Authors:** Apoorva Bharthur Sanjay, Li Sun, Deepanshi Shokeen, Ole Isacson, So‐Youn Shin, Jeanne Latourelle

**Affiliations:** ^1^ Aitia, Somerville, MA USA; ^2^ Harvard Medical School / McLean Hospital, Belmont, MA USA

## Abstract

**Background:**

Amyloid, Tau and neurodegeneration (ATN), the hallmark pathologies of Alzheimer’s Disease (AD) translating to measurable biomarkers are important for disease modifying therapeutics.

**Method:**

AD Digital‐Twins were built using AITIA’s patented A.I. platform REFS^TM^ [aitiabio.com], based on a Bayesian network model of ADNI data which reverse‐engineered the connectivity of ∼59K multi‐modal variables and AD‐related outcomes profiled from 317 subjects (Control: MCI: Dementia=97:191:29). The average causal effect of each upstream‐downstream variables was estimated through in‐silico counterfactual experiments to evaluate the temporal relationship between the ATN outcomes, to identify the causal gene‐drivers (at blood RNA‐expression level) of “ATN” outcomes, and to investigate the known AD genotypic variants strongly driving ATN gene‐drivers. Age‐gene interaction was additionally explored through “double‐intervention” experiments, to evaluate age‐specific effects of gene‐drivers on ATN outcomes.

**Result:**

AD Digital‐Twins evaluated the ATN temporal relationship, and recapitulated some known relationships such that CSF‐abeta and Tau measures drive neurodegeneration measures, although it also showed CSF‐Tau measures to be upstream of amyloid PET, suggesting Tau changes may interact with other outcome changes more dynamically over the course of disease progression (Figure 1). Next, in‐silico experiments identified a total of 228 ATN gene‐drivers, including 8 common to all outcome‐categories, which affect cognitive outcomes as well (Figure 2) and are strongly related to immune‐response and inflammation pathways. Many of these genes were causally driven by multiple AD‐associated genotypic variants reported in GWAS, especially in the “NECTIN2” and “APOE” region. Furthermore, in‐silico experiments showed some Tau‐driving genes are likely to be causally driven by Amyloid and Neurodegeneration driving genes. Lastly, age‐specific effects were observed for a portion of each A‐, T‐ and N‐ gene‐drivers, especially with Tau‐driving genes having a stronger effect with opposite directionality for younger vs older age groups (Figure 3).

**Conclusion:**

Tau related abnormalities are likely early events in AD progression and more strongly linked to disease pathophysiology. Aitia’s AI platform allows powerful and systematic evaluation of multiple modalities and outcomes, accelerating precision medicine efforts in AD.